# Proof of Concept Study: Comparison of Semi-Automated RNA Isolation Methods from Archived Formalin-Fixed, Paraffin-Embedded Tissues with Clinical Routine RNA Isolation Methods

**DOI:** 10.3390/mps7060101

**Published:** 2024-12-19

**Authors:** Patrick Hannibal Dalsbo Petersen, Jaslin Pallikkunnath James, Lene Buhl Riis, Claus Kim Høgdall, Estrid Vilma Høgdall

**Affiliations:** 1 Department of Pathology, Herlev University Hospital, 2730 Herlev, Denmark; 2 Department of Gynecology, The Juliane Marie Centre, Rigshospitalet, University of Copenhagen, 2100 Copenhagen, Denmark; claus.hogdall@regionh.dk

**Keywords:** FFPE, RNA extraction, KingFisher, automation, AutoLys, deparaffinization, xylene, limonene

## Abstract

High-quality RNA is crucial in clinical diagnostics and precision medicine. Formalin-fixed and paraffin-embedded (FFPE) tissues pose a challenge due to nucleic acid fragmentation and crosslinking. In this pilot study, various commercially available techniques for extracting RNA from small FFPE samples were compared. We evaluated the KingFisher Duo automated system or the manual MagMAX FFPE DNA/RNA Ultra Kit as an RNA extraction method combined with either a xylene, d-limonene, or AutoLys M tubes deparaffinization method. Additionally, the automated Maxwell RSC RNA FFPE kit and the High Pure FFPET RNA Isolation Kit were examined using FFPE samples from inflammatory bowel disease (IBD) patients, as well as samples from ovarian, kidney, and breast cancer and the skin. The KingFisher Duo system gave a higher yield and more consistent RNA quantities, especially from small volumes of IBD samples, compared to manual extraction. The deparaffinization method also impacted results, with AutoLys M tubes proving effective in combination with the KingFisher Duo system. Conversely, the High Pure kit exhibited higher yields for larger FFPE samples. While RNA integrity is a critical factor, particularly for messenger RNA (mRNA) expression studies, its role is less prominent in microRNA (miRNA) analyses. Recognizing this, our study focused on RNA yield and purity (A260/A230) to evaluate RNA extraction methods for various sample types. These findings emphasize the importance of selecting appropriate RNA extraction methods based on sample characteristics and research goals, highlighting the performance of automated methods and the impact of deparaffinization choices. The findings contribute to refining RNA extraction for molecular biology analyses, suggesting avenues for further exploration, including cost-effectiveness under specific experimental conditions.

## 1. Introduction

In the field of clinical diagnostics and treatment planning emphasizing precision medicine, the procurement of high-quality nucleic acids, such as RNA, holds paramount importance, as downstream analyses may impact treatment decisions for patients. These materials serve as the foundation for a wide range of diagnostic assays and analyses, including higher-end techniques such as next-generation sequencing (NGS) and gene expression profiling, enabling comprehensive insights into disease processes that impact diagnostics and guide personalized treatment approaches [1,2,3].

Formalin-fixed and paraffin-embedded (FFPE) tissues have been widely used in clinical settings for decades. Their stability over extended periods, ease of storage, ability to preserve histopathology, scope for biobanking, availability as historical samples, and cost-effectiveness make FFPE tissues an invaluable tool for clinical research and diagnostic applications [4,5,6,7]. Moreover, several centers across the globe have developed huge archives of FFPE tissue samples for various clinical specimen types, which are connected to clinical and follow-up databases for the purpose of translational research [8,9,10,11]. In Denmark, a Nationwide clinical biobank, the Bio- and GenomeBank, Denmark, was established for the optimal use of biological material for clinics, as well as for translational research.

However, the fixation and embedding processes of FFPE tissues introduce significant challenges to RNA extraction. Formalin fixation induces extensive crosslinking between RNA and proteins, resulting in fragmentation and contamination, which complicates downstream applications like qPCR and NGS. This is particularly challenging in small samples like needle biopsies, where paraffin content is disproportionately higher. Reversing these modifications and ensuring high-quality RNA requires robust deparaffinization protocols and precise RNA isolation methods.

To revert crosslinking, FFPE tissues need to be deparaffinized. This has traditionally been performed using xylene (dimethyl benzene), a carcinogenic aromatic hydrocarbon [12,13,14]. Though many commercial RNA extraction methods have been released, peer reviewed evaluations are still lacking [15].

In our experience, extracting high-quality RNA from small FFPE samples remains a challenge for both automatic and manual RNA extraction methods. While automatic platforms offer efficiency, they often struggle with low RNA yields and contaminant removal. On the other hand, manual isolation can achieve better purity but requires significant time and labor as well as the handling of hazardous chemicals, raising safety concerns. These limitations are particularly pronounced in the context of inflammatory bowel disease (IBD), which describes two conditions characterized by chronic inflammation of the gastrointestinal tract. These biopsies, usually 1–2 mm^3^ in size, are stored as FFPE, presenting a challenge for RNA extraction due to the larger amounts of paraffin and lower gene expression compared to cancerous tissues.

We conducted a comparative analysis of various commercial techniques to identify an effective and efficient RNA extraction workflow, particularly for small FFPE samples (e.g., needle biopsies). Our objective with this proof of concept was to establish a practical approach that optimizes both the quantity and quality of RNA while minimizing the exposure to hazardous chemicals. Therefore, we specifically focused on the challenges of RNA extraction from archived FFPE needle biopsies from IBD patients, and furthermore, we also compared the RNA yield and purity of the extracted RNA to that obtained from larger FFPE tissue biopsies from breast and kidney tumors, the skin, and a fresh frozen ovarian cancer tumor. The samples used in this study were collected with the intent of being used for research purposes.

## 2. Materials and Methods

### 2.1. Study Design

**Figure 1:**
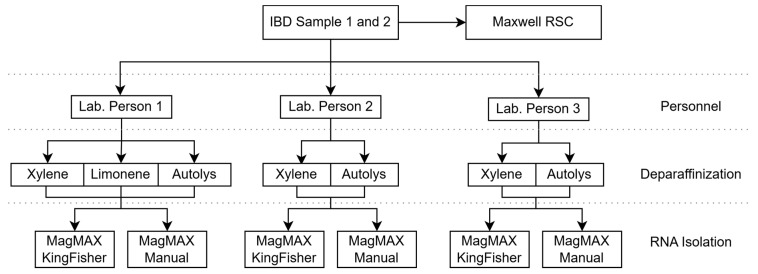
Workflow diagram depicting the initial study setup. Two inflammatory bowel disease colon FFPE samples were utilized to explore RNA isolation using the MagMAX FFPE DNA/RNA Ultra Kit (Thermo Fisher Scientific), either manually or semi-automatically, using a KingFisher Duo prime Magnetic Particle Processor (Thermo Scientific, Life Technologies, Singapore). Xylene, d-limonene, and AutoLys M tubes were used for deparaffinization. RNA was additionally isolated using a semi-automatic Maxwell^®^ RSC RNA FFPE kit on a Maxwell RSC instrument (Promega Corporation, Madison, WI, USA).

In this study, three experienced laboratory personnel performed RNA extraction on two 10 µm sections from FFPE blocks, each with an area of approximately 1–2 mm^2^ of tissue. The MagMAX FFPE DNA/RNA Ultra Kit from Thermo Fisher Scientific (Thermo Fisher Scientific, Roskilde, Denmark) was employed for total RNA extraction, including microRNAs. Deparaffinization was carried out manually using xylene, d-limonene, or AutoLys M tubes, followed by either manual or automated RNA extraction. Additionally, the commonly used RNA extraction method in routine practice at the department, Promega’s Maxwell RSC RNA FFPE kit (Nordic Biolabs, Täby, Sweden), was included for comparison using the tissue slides from the same blocks. Total RNA quality and quantity were assessed using Nanodrop and Qubit instruments. An overview of the study plan is depicted in Figure 1.

### 2.2. Samples

This study utilized three sets of samples. The initial set comprised FFPE samples, known for their challenging RNA extraction, and included two IBD samples, specifically chosen for their small area (10–15 mm^2^) and notably high paraffin-to-tissue ratios. The second set comprised five FFPE samples, featuring larger tissue fragments from kidney and breast cancer tumors and from the skin. These samples were larger (approximately 2–3 cm^2^), resulting in a significantly smaller fraction of paraffin as compared to the first set. Finally, a set of fresh frozen high-grade serous ovarian cancer tumor samples was utilized, representing high-quality tissue specimens (see Table 1).

### 2.3. RNA Extraction

Five different protocols were used for RNA isolation. An overview of the main steps can be seen in Table 2.

*Promega semi-automatic RNA extractions* were performed on two 10 µm FFPE slices submerged in 300 µL of mineral oil using the Maxwell^®^ RSC RNA FFPE Kit (Promega, Madison, WI, USA, Cat: AS1440) on a Maxwell^®^ RSC Instrument (Promega) according to the manufacturer’s protocol [16]. The RNA was eluted in 55 µL of Tris-EDTA buffer.

*Roche manual RNA extractions* were performed on two 10 µm FFPE slices prepared on two microscope slides and incubated at 60 °C for 45 min. RNA was isolated from these using the High Pure FFPET RNA Isolation Kit (Roche, Basel, Switzerland, Cat.: 06650767001) according to the manufacturer’s protocol, except d-limonene was used as a clearing agent instead of xylene [17]. RNA was eluted in 30 µL of elution buffer, added to the High Pure filter, and incubated for 1 min prior to a one-minute centrifugation at 16,000× *g*. Potential unwanted debris was then pelleted by 2 min of centrifugation and removed from the supernatant.

*Thermo manual RNA extractions* were performed on two 10 µm of RNA from FFPE samples using the MagMAX FFPE DNA/RNA Ultra Kit (Thermo Fisher Scientific, Roskilde, Denmark, Cat.: A31881) according to the manufacturer’s protocol [18,19]. Deparaffinization was performed using either d-limonene or xylene, according to the manufacturer’s protocol [18], or via AutoLys M tubes (Thermo Fisher Scientific, Roskilde, Denmark, Cat.: A38738), according to the manufacturer’s protocol [19]. RNA was eluted in 50 µL of elution buffer in the shaker at 1150 rpm before the magnets were removed and the eluate collected.

*Thermo semi-automatic RNA extractions* were performed on two 10 µm of RNA from FFPE samples in a KingFisher Duo prime Magnetic Particle Processor (Thermo Scientific, Life Technologies, Singapore, Cat.: 5400110) using the MagMAX FFPE DNA/RNA Ultra Kit (Thermo Fisher Scientific, Roskilde, Denmark, Cat.: A31881) according to the manufacturer’s protocol [18,19]. Deparaffinization was performed as described above for the Thermo manual RNA extraction. RNA was eluted in 50 µL of elution buffer.

*Fresh frozen tissue samples*—about 5 mg of tissue was homogenized in 110 µL of lysis binding mix using a micro pestle on a Micro-Grinder pestle mixer for 30 s in a 1.5 mL reaction tube. The isolation was performed via the KingFisher Duo Prime Magnetic Particle Processor (Thermo Fisher Scientific, Cat.: 5400110) using the MagMAX mirVana Total RNA Isolation Kit (Thermo Fisher Scientific, Roskilde, Denmark, Cat.: A27828) according to the manufacturer’s protocol [20]. Finally, RNA was eluted in 75 µL of elution buffer.

All RNA samples were stored at −20 °C for a maximum of 48 h before being evaluated.

**Table 2:** Comparison of RNA extraction methods.

	Technology	Protocol Used	Preparation of Tissue	Deparaffinization	Tissue Lysis and Proteinase Treatment	DNAse Treatment	RNA Elution	Overall Time of Protocol	Hands-On Time
Roche column-based	Manual	High Pure FFPET RNA Isolation Kit ([17])	Two of the 10 µm FFPE tissue slices were prepared on microscopic slides	D-Limonene	Proteinase K in lysis buffer	DNase I DNase buffer	TE-buffer	~16–24 h	~2 h
Promega magnetic bead-based	Semi-automatic	Maxwell^®^ RSC RNA FFPE Kit ([16])	Two of the 10 µm FFPE tissue slices	Mineral oil	Proteinase K in lysis buffer	DNase I DNase buffer	Performed on a Maxwell RSC instrument (Promega); RNA was eluted in TE buffer (~40 min)	~16–24 h	~1 h
Thermo magnetic bead-based	Manual	MagMAX FFPE DNA/RNA Ultra Kit ([18])	Two of the 10 µm FFPE tissue slices	Protease K in AutoLys M tubes		DNase I DNase buffer	Elution solution	~4 h	~1–2 h
				Xylene	Lysis buffer proteinase K			~4–5 h	~2 h
				D-Limonene					
	Semi-automatic			Protease K in AutoLys M tubes		Performed on a KingFisher Duo instrument (Thermo); RNA was eluted in an elution solution (~50 min)		~4 h	~30 min
				Xylene	Lysis buffer proteinase K			~4–5 h	~1 h
				D-Limonene					

### 2.4. RNA Quality Control

RNA concentrations, as well as the purity and contamination ratio values, (A260/A230 and A260/A280 respectively), were measured on a NanoDrop One (ThermoFisher Scientific, Waltham, MA, USA, Cat.: 701-058112).

### 2.5. Data Analysis

All data analyses and graph generations were performed using R Statistical Software version 4.3.2 [21] and R studio IDE version 2023.12.1 [22]. A descriptive analysis methodology was adopted for this study, with mean values employed for group comparisons.

## 3. Results

### 3.1. Comparison of Manual and Automatic Isolation of IBD FFPE Samples

**Figure 2:**
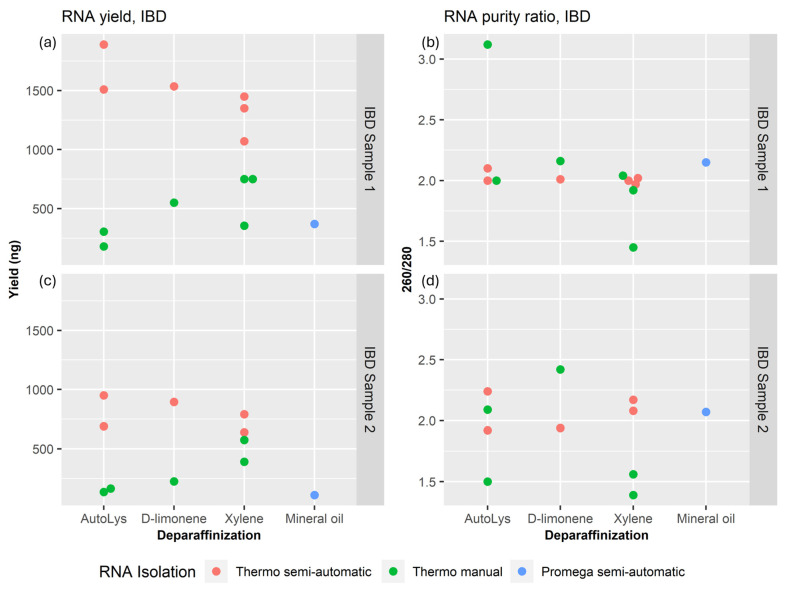
Comparison of yield and purity measured using a Nanodrop One (Thermo Fisher Scientific) of isolated RNA from two small inflammatory bowel disease (IBD) FFPE samples. (**a**) Total RNA yields isolated from IBD sample 1. (**b**) Purity of RNA isolated from IBD sample 1. (**c**) Total RNA yields isolated from IBD sample 2. (**d**) Purity of RNA isolated from IBD sample 2. Three isolation methods were employed: MagMAX FFPE DNA/RNA Ultra Kit from Thermo Fisher Scientific, manual (referred to as Thermo manual) or semi-automatic (referred to as Thermo semi-automatic) with a KingFisher Duo prime Magnetic Particle Processor (also from Thermo Fisher Scientific), and Maxwell^®^ RSC RNA FFPE Kit from Promega (referred to as Promega semi-automatic). Different deparaffinization methods were applied for the Thermo MagMAX isolation: xylene, d-limonene, or AutoLys M tubes from Thermo Fisher Scientific.

The Thermo semi-automatic RNA extractions consistently provided higher yields of between 995 ng and 1890 ng for IBD sample 1 and between 730 ng and 982 ng for IBD sample 2 (Figure 2a,c, red dots). The mean RNA yield for the KingFisher semi-automatic method was 1216.6 ng with a standard deviation (STD) of 433.6 ng for IBD sample 1 and 845.2 with a STD of 103.5 ng for IBD sample 2. This is consistently above the yield provided by the Thermo manual RNA extractions, which yielded between 268 ng and 694 ng with a mean of 528.0 ng and a STD of 180.5 ng for IBD sample 1, and between 210 ng and 509 ng for IBD sample 2 (Figure 2a,c, green dots). Two manual isolations, using AutoLys and xylene, did not provide measurable yields. Statistical analysis revealed that the difference in RNA yields between the KingFisher semi-automatic method and the manual method was highly significant (*p* = 2.36 × 10^−6^).

RNA purity ratios were generally better for the Thermo semi-automatic isolations. These were all between 1.97 and 2.02 (mean = 2.00, STD = 0.05) for IBD sample 1, and between 1.92 and 2.24 (mean = 2.07, STD = 0.13) for IBD sample 2 (Figure 2b,d; red dots). Thermo manual RNA extractions had more variation in purity ratios. These were between 1.45 and 3.12 for IBD sample 1 and between 1.39 and 2.42 for IBD sample 2 (Figure 2b,d; green dots). Despite this variability, statistical comparison of the purity ratios (A260/A280) between the two methods did not show a significant difference (*p* = 0.65). The Promega semi-automatic RNA extraction on IBD sample 1 isolation resulted in a yield on the lower end (435 ng) and a purity ratio of 2.15 (Figure 2, blue dots).

### 3.2. Comparison of Deparaffinization Methods Using IBD FFPE Samples

In the case of Thermo manual RNA extractions (Figure 2), optimal average yields were obtained using xylene as the deparaffinization agent for both sample 1 (618.33 ng) and sample 2 (482.5 ng). Conversely, d-limonene yielded 550 ng for sample 1 and 225 ng for sample 2, while the AutoLys tubes provided the lowest yields, averaging 242.5 ng for sample 1 and 150 ng for sample 2, whereas the results were inverted for Thermo semi-automatic RNA extractions. AutoLys tended to be the more effective deparaffinization agent, yielding an average of 1700 ng for sample 1. On the other hand, d-limonene and xylene exhibited averages of 1535 ng and 1290 ng, respectively, for sample 1. The differences were less pronounced for sample 2, where d-limonene yielded a slightly higher amount than the AutoLys average (895 ng compared to 820 ng), with xylene providing the lowest average yield of 715 ng.

### 3.3. Large Tissue FFPE Samples

**Figure 3:**
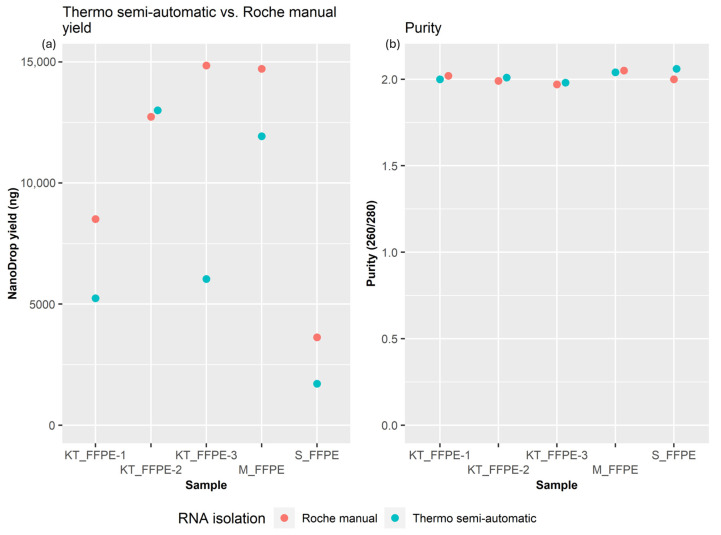
Comparison of (**a**) total RNA yields and (**b**) purity of the RNA isolated from large FFPE tissues from a kidney tumor (KT), a mammary tumor (M), and the skin (S) performed using KingFisher Duo RNA isolation (referred to as Thermo semi-automatic) and High Pure manual isolation (referred to as Roche manual). Yield (**left**) and purity ratios (**right**) measured using a Nanodrop One (Thermo Fisher Scientific).

When testing the second set of larger tissue FFPE samples (Table 1), the best manual routine extraction method, Roche manual RNA extraction, outperformed Thermo semi-automatic RNA extraction in four out of five samples, with the last sample showing similar results for both methods. Roche manual RNA extraction consistently provided a higher RNA yield, with a mean of 10,889 ng, compared to 7583 ng for the Thermo semi-automatic RNA extraction. On average, Roche provided approximately a 3300 ng greater yield (Figure 3), although this difference was not statistically significant (*p* = 0.093) based on a paired t-test analysis. The A260/A280 values from the NanoDrop were similar and acceptable (ranging from 1.97 to 2.05) for all samples. Regarding the contamination ratio, Roche Manual RNA extraction consistently produced better results, with values ranging from 1.66 to 2.24, while the Thermo semi-automatic RNA extraction values were consistently below 1.5.

### 3.4. Fresh Frozen Tissue with KingFisher Duo

**Figure 4:**
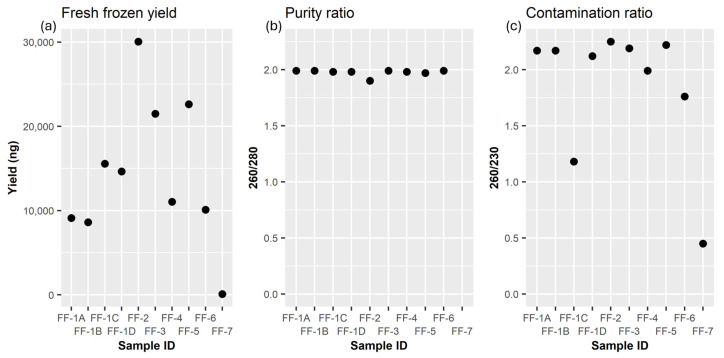
RNA from fresh frozen ovarian cancer tumor tissue. (**a**) Yield, (**b**) purity ratio, and (**c**) contamination ratio measured using a Nanodrop One (Thermo Fisher Scientific) on RNA extracted from fresh frozen ovarian cancer tissue. RNA was extracted using the MagMAX mirVana Total RNA Isolation Kit (Thermo Fisher Scientific) on the KingFisher Duo prime Magnetic Particle Processor (Thermo Fisher Scientific).

As a benchmark for optimal performance, we also extracted RNA from fresh frozen ovarian cancer samples. Using the KingFisher Duo for RNA isolation from these high-grade serous adenocarcinoma tissues yielded amounts ranging from 8600 ng to 30,060 ng, in most cases. Consistent outcomes were observed in repeated samples (Figure 4, samples FF-1A through FF-1D), particularly evident in the A260/A280 values, maintaining purity ratios consistently between 1.97 and 1.99.

Overall, the A260/A280 values indicated satisfactory results, except for sample FF-7, which exhibited both low yield and purity (Figure 4b). In contrast to FFPE extractions, the fresh frozen samples demonstrated A260/A230 ratios that were generally closer to the optimal range. Among the ten extractions, six fell within the range of 2.0 to 2.2, two were below this range at 0.45 and 1.18, and two were above at 2.22 and 2.25.

### 3.5. Cost Analysis

To further support practical decision-making in routine laboratory settings, we evaluated the approximate costs of each method based on Danish laboratory technician salaries and kit prices. For processing eight samples, the Thermo manual method totaled $120.75 (including $56.43 in labor and $64.33 in kit costs), while the Thermo automatic method cost approximately $71.38 (with only $7.05 in labor due to reduced hands-on time). The Maxwell method totaled $120.75 ($28.21 labor and $92.54 kit costs), and the High Pure automatic method cost $101.57 ($56.43 labor and $45.14 kit costs). Equipment investment also plays a role in cost considerations; for example, the KingFisher Duo Prime instrument required for the Thermo automatic method has an estimated cost of $47,300, and the Promega Maxwell RSC instrument costs around $58,627. While these up-front costs can represent a significant investment, automated methods may reduce long-term costs through labor savings and increased throughput, which could be especially beneficial for high-volume laboratories.

## 4. Discussion

In this proof of concept, we evaluated manual and semi-automatic RNA isolation methods and different deparaffinization protocols to assess their impact on RNA yield and purity. The samples analyzed were representative of the diverse biological materials processed in a pathologydepartment. Although the sample size was limited, making statistical conclusions preliminary, the findings provide valuable insights into optimizing RNA extraction protocols for future research.

The challenges associated with FFPE sample preparation were evident, particularly for small samples like inflammatory bowel disease (IBD) needle biopsies. The fixation process causes RNA fragmentation and crosslinking, while the presence of paraffin exacerbates contamination, complicating downstream applications, such as RNA sequencing and qPCR. Small FFPE samples amplify these challenges, as limited material availability requires careful optimization to maximize RNA yield and purity.

Our findings revealed statistically significant differences in RNA yield (*p* = 2.36 × 10^−6^), emphasizing the importance of method selection. The Thermo KingFisher Duo automated system with the MagMAX FFPE DNA/RNA Ultra Kit consistently produced higher yields compared to manual methods, averaging 1216.6 ng (STD 433.6) for IBD sample 1 and 845.2 ng (STD 103.5) for IBD sample 2.

The Promega semi-automatic method used routinely in the laboratory exhibited lower yields. These results align with findings by Schmeller et al., who reported that the Promega RNA extraction method provided better RNA quality but at a lower total yield compared to a manual Qiagen RNeasy FFPE kit, highlighting the trade-off between yield and quality across different methods [14].

Manual methods, while achieving occasional improvements in purity, showed significant variability and required substantial labor and hazardous chemical handling. This highlights the trade-off between automation and manual workflows, where automated methods like KingFisher Duo are better suited for high-throughput environments requiring a consistent purity and yield.

Xylene, a traditional clearing agent used in deparaffinization protocols, has long been considered the gold standard for removing paraffin from FFPE tissue [14,23]. However, its association with adverse health effects, including cancer, respiratory complications, and neurological disorders, raises concerns regarding its long-term use [12,13]. In this study, the use of d-limonene as a substitute for xylene did not lead to significant differences in RNA yield or purity, reinforcing the potential of d-limonene as a safer alternative to xylene. This finding aligns with the work of Schmeller et al., who also reported that d-limonene yields results comparable to traditional clearing agents, like oil and xylene, in RNA extraction from FFPE tissues [14]. AutoLys M tubes in combination with the KingFisher Duo tended to perform better as compared to manual methods with less hands-on and overall time requirements.

The RNA yields observed in our study ranged from 210 ng to 1890 ng for small 10 µm IBD samples, and from 1710 ng to 14,855 ng for larger FFPE samples. These results are comparable to our previous findings, where an average yield of 5200 ng was obtained from medium-sized FFPE sections of ovarian cancer samples of similar thicknesses [9]. Moreover, with advancements in RNA sequencing technologies, such as the TruSeq kit and the SMARTer series (including SMARTer and SMARTer Ultra-Low from Takara Bio/Clontech), RNA inputs as low as 100 ng—and even below 1 ng for the SMARTer Ultra-Low—are adequate for successful sequencing [24]. This indicates that the RNA yields from both xylene and d-limonene preparations are more than sufficient for these downstream applications.

While yield is an important factor for downstream analysis, low concentration samples can be up-concentrated by, for example, vacuum centrifugation, ethanol precipitation, or column-based re-isolation [25,26]. This has been demonstrated to impact successful analyses performed in routine settings, if material is limited. However, if obtaining the utmost quality and quantity in the initial workflow is paramount, up-concentration is still feasible, recognizing that additional procedures may potentially prolong the time required for a diagnostic analytical response.

Purity ratios, represented by the A260/A280 and A260/A230 values, are important indicators of RNA quality. The optimal A260/A230 ratio for pure RNA is between 2.0 and 2.2; lower values may indicate contaminants like EDTA, carbohydrates, and phenol [27]. As seen in Figure 2, deparaffinization methods showed minimal influence compared to RNA isolation methods. IBD sample 1 consistently yielded better results, reflecting its higher concentration, which mitigates the impact of small variations in absorbency on the A260/A280 ratio. Compared to automatic isolations (Figure 2), manual extractions resulted in decreased yields and an increased variability in purity (Figure 2).

This study exclusively evaluated RNA yield and purity. However, it is important to acknowledge that RNA integrity, the preservation of full-length RNA molecules, is another crucial factor for downstream applications. While techniques like gel electrophoresis, microfluidic analysis, and the calculation of the ribosomal RNA ratio (RIN) are valuable for assessing messenger RNA (mRNA) integrity, their application to microRNA (miRNA) analysis, particularly from FFPE tissues, is less informative. Except for the Promega semi-automatic RNA extraction, which yielded values of 2.15 for IBD sample 1 and −17.67 for IBD sample 2, all protocols demonstrated values ranging between 0.01 and 0.55, which aligns with our typical experience of RNA extraction from FFPE samples. The outlier value observed for IBD sample 2 could be attributed to its low concentration. Minor fluctuations in sample contaminants could lead to significant variations in the ratios between RNA and potential contaminants. While differences were minor, A260/A230 values for automated isolations consistently exceeded those of manual isolations (Figure S1). Despite variations, for the A260/A230 values, automated isolations consistently exceeded those of manual isolations, indicating a potential advantage in reducing contaminants.

Comparing the Promega semi-automatic RNA extraction, Roche manual RNA extraction, and Thermo semi-automatic RNA extraction methods, we observed trade-offs between yield and purity. While manual isolations yielded the highest RNA quantities, they exhibited lower purity ratios. In contrast, Promega semi-automatic RNA extraction maintained relatively stable purity ratios, despite lower yields. Thermo semi-automatic RNA extraction samples showed a yield–purity ratio fluctuation between days.

In the context of large tissue FFPE samples, the Roche manual RNA extraction method outperformed the Thermo semi-automatic RNA extraction method in terms of RNA yield. The consistent superiority of Roche manual RNA extraction in four out of five samples underscores its reliability for larger tissue specimens and suggests that several extraction methods should be considered in a routine laboratory setting that uses different sample types. Purity ratios further supported the advantage of Roche manual RNA extraction, with consistently better values compared to Thermo semi-automatic RNA extraction.

For fresh frozen tissue samples used in this study, the KingFisher Duo demonstrated consistent and acceptable results, particularly in the A260/A280 values, that are comparable to the A260/A280 values obtained from the large tissue FFPE samples. These results for the fresh frozen tissue were also comparable to other methods, like the miRNeasy Tissue/Cells Advanced Micro Kit from Qiagen and the SV Total RNA Isolation System from Promega [9,28], emphasizing the negative effects of paraffin in small sample FFPE tissues.

The Roche manual RNA extraction protocol excels in providing thorough isolation with the use of d-limonene but is time-consuming and labor-intensive. The Promega semi-automatic RNA extraction protocol stands out for its speed and low hands-on requirements but may lack explicit information about d-limonene use. The Thermo semi-automatic RNA extraction, with the option to use AutoLys M tubes, presents a compromise by decreasing labor and time, although there might be a delay due to manual filling in certain setups. The choice among these protocols should consider the specific requirements of the analysis, emphasizing factors such as time, labor, and the desired level of RNA isolation. Of the methods used here, the Roche manual RNA extraction was the most time-consuming, requiring the preparation of tissues onto slides, overnight heat incubation, and column-based isolation. The Promega semi-automatic RNA extraction protocol was the fastest with the least hand-on requirements. Though the incubation times are identical, exchanging the xylene or d-limonene in the deparaffinization step in the MagMAX protocol with the AutoLys M tubes represented a marked decrease in manual labor and time consumption. Due to the manual filling of the KingFisher 96 Deep-Well Plate, the Thermo semi-automatic RNA extraction protocol takes longer than that for Promega semi-automatic RNA extraction. For troubleshooting and optimization strategies related to this protocol, please refer to the Supplementary Materials (Table S1).

Cost analysis indicated that automated methods offer long-term efficiency despite higher initial investment costs for instruments. For example, the Thermo KingFisher Duo system had lower hands-on labor costs, and a higher throughput capacity compared to manual workflows. However, the choice of protocol should balance cost, labor requirements, and downstream application needs, particularly in routine diagnostic settings, where efficiency and reliability are critical.

When comparing manual and automated methods, a clear trade-off emerges. Automated methods, such as the KingFisher Duo, deliver higher yields and consistent purity, making them suitable for high-throughput environments. Conversely, manual methods occasionally achieve higher purity ratios but exhibit significant variability, require substantial labor, and necessitate hazardous chemical handling. The choice of method must be informed by the specific downstream requirements, balancing yield, purity, and throughput needs.

For challenging samples, like FFPE needle biopsies, optimizing both RNA yield and purity is essential to ensure diagnostic reliability. Future studies should explore reagent combinations or additional purification steps to address residual contaminants in automated protocols.

## 5. Conclusions

In conclusion, this assessment of RNA extraction methods underscores the importance of selecting appropriate techniques based on sample characteristics. Automated methods consistently outperformed manual ones across various scenarios assessed in this study using small FFPE biopsies. Notably, for manual implementation of the MagMAX protocol, the use of xylene or d-limonene proved preferable to AutoLys tubes. Conversely, in automated protocols, AutoLys tubes demonstrated superiority over other methods used in this study. Fresh Frozen tissue was found to be the optimal tissue for RNA extraction, with consistently high yield and purity, and may, if possible, be preferred for downstream molecular analysis. Further investigations could delve into additional factors affecting method performance, such as cost-effectiveness and scalability, providing valuable guidance to laboratories in choosing the most, or several, suitable approaches for specific experimental conditions.

In conclusion, our study contributes to the importance of refinement of RNA extraction protocols, ensuring dependable applications in molecular biology research and increasingly comprehensive routine workflows.

## Figures and Tables

**Table 1:** Properties and characteristics of the three sample sets included in the study. Set 1 consisted of small FFPE colon samples from inflammatory bowel disease patients. Set 2 consisted of large FFPE samples from kidney tumors, mammary tumors, and the skin. Set 3 consists of freshly frozen high-grade serous ovarian cancer tumor samples. Approximate exposed area is given for FFPE samples and approximate mass is given for fresh frozen samples.

Sample	Age	Tissue Size (Area/Mass)	Format	Fixation Time
**Sample set 1**				
IBD-1	~2 years	~10–15 mm^2^	FFPE	NA
IBD-2	~2 years	~10–15 mm^2^	FFPE	NA
**Sample set 2**				
S_FFPE	<1 year	~2–3 cm^2^	FFPE	48 h
KT_FFPE-1	<1 year	~2–3 cm^2^	FFPE	>48 h
KT_FFPE-2	3 years	~2–3 cm^2^	FFPE	>48 h
M_FFPE	<1 year	~2–3 cm^2^	FFPE	>48 h
KT_FFPE-3	3 years	~2–3 cm^2^	FFPE	>48 h
**Sample set 3**				
OC_FF-1A		~5–6 mg	Fresh Frozen	NA
OC_FF-1B		~5–6 mg	Fresh Frozen	NA
OC_FF-1C		~5–6 mg	Fresh Frozen	NA
OC_FF-1D		~5–6 mg	Fresh Frozen	NA
OC_FF-2		~5–6 mg	Fresh Frozen	NA
OC_FF-3		~5–6 mg	Fresh Frozen	NA
OC_FF-4		~5–6 mg	Fresh Frozen	NA
OC_FF-5		~5–6 mg	Fresh Frozen	NA
OC_FF-6		~5–6 mg	Fresh Frozen	NA
OC_FF-7		~5–6 mg	Fresh Frozen	NA

IBD: inflammatory bowel disease, S: skin, KT: kidney tumor, M: mammary tumor, OC: ovarian cancer, NA: not available.

## Data Availability

All relevant data are within the paper and its Supplementary Materials.

## Supplementary Materials

The following supporting information can be downloaded at: https://www.mdpi.com/article/10.3390/mps7060101/s1, Figure S1: RNA A260/A230 contamination ratio values for IBD samples; Table S1: Troubleshooting guide for RNA extraction from FFPE tissues.
