# Supplementary material for: Proof of Concept Study: Comparison of Semi-Automated RNA Isolation Methods from Archived Formalin-Fixed, Paraffin-Embedded Tissues with Clinical Routine RNA Isolation Methods
Source: Methods Protoc. 2024 Dec 19;7(6):101. doi: 10.3390/mps7060101 (PMC11678837; doi:10.3390/mps7060101)
Supplement: Supplementary file 1 [file mps-07-00101-s001.zip › Supplemental Table S1.pdf]

Table S1: Troubleshooting Guide for RNA Extraction from FFPE Tissues.

Issue	Possible Cause	Solution
Low RNA Yield	Insufficient tissue amount or poor tissue quality.	Ensure that the FFPE samples contain sufficient tissue for extraction. Check the quality of the tissue sections before starting the protocol. If yield remains low, consider increasing the number of tissue sections.
RNA Degradation	Prolonged exposure to heat or reagents during deparaffinization.	Minimize the exposure time to xylene or d-limonene and avoid excessive heating during the deparaffinization and rehydration steps. Use RNase-free reagents and equipment to prevent contamination
Poor RNA Purity (Low A260/A280 or A260/A230 Ratios)	Incomplete removal of d-limonene or xylene, or contamination with proteins and phenol.	Ensure thorough washing steps following deparaffinization to remove all traces of d-limonene or xylene. Use additional ethanol washes if necessary.
Contamination Detected in RNA (e.g., DNA or Protein Contaminants)	Incomplete removal of contaminants during extraction.	Incorporate additional DNase treatment to eliminate DNA contamination. Ensure proper proteinase K digestion.
Difficulty in Deparaffinization Using d-Limonene	Inadequate incubation time or temperature.	Optimize the incubation time and temperature for d-limonene to ensure complete paraffin removal. Longer incubation or slightly increased temperature may improve deparaffinization efficiency
Inhibition in Downstream Applications (e.g., RT-qPCR, RNAseq)	Residual contaminants or inhibitors in the RNA preparation	Perform an additional purification step, such as a second ethanol precipitation or column-based cleanup, to remove potential inhibitors before proceeding to downstream applications.
