# Supplementary figures and images for: Proof of Concept Study: Comparison of Semi-Automated RNA Isolation Methods from Archived Formalin-Fixed, Paraffin-Embedded Tissues with Clinical Routine RNA Isolation Methods
Source: Methods Protoc. 2024 Dec 19;7(6):101. doi: 10.3390/mps7060101 (PMC11678837; doi:10.3390/mps7060101)

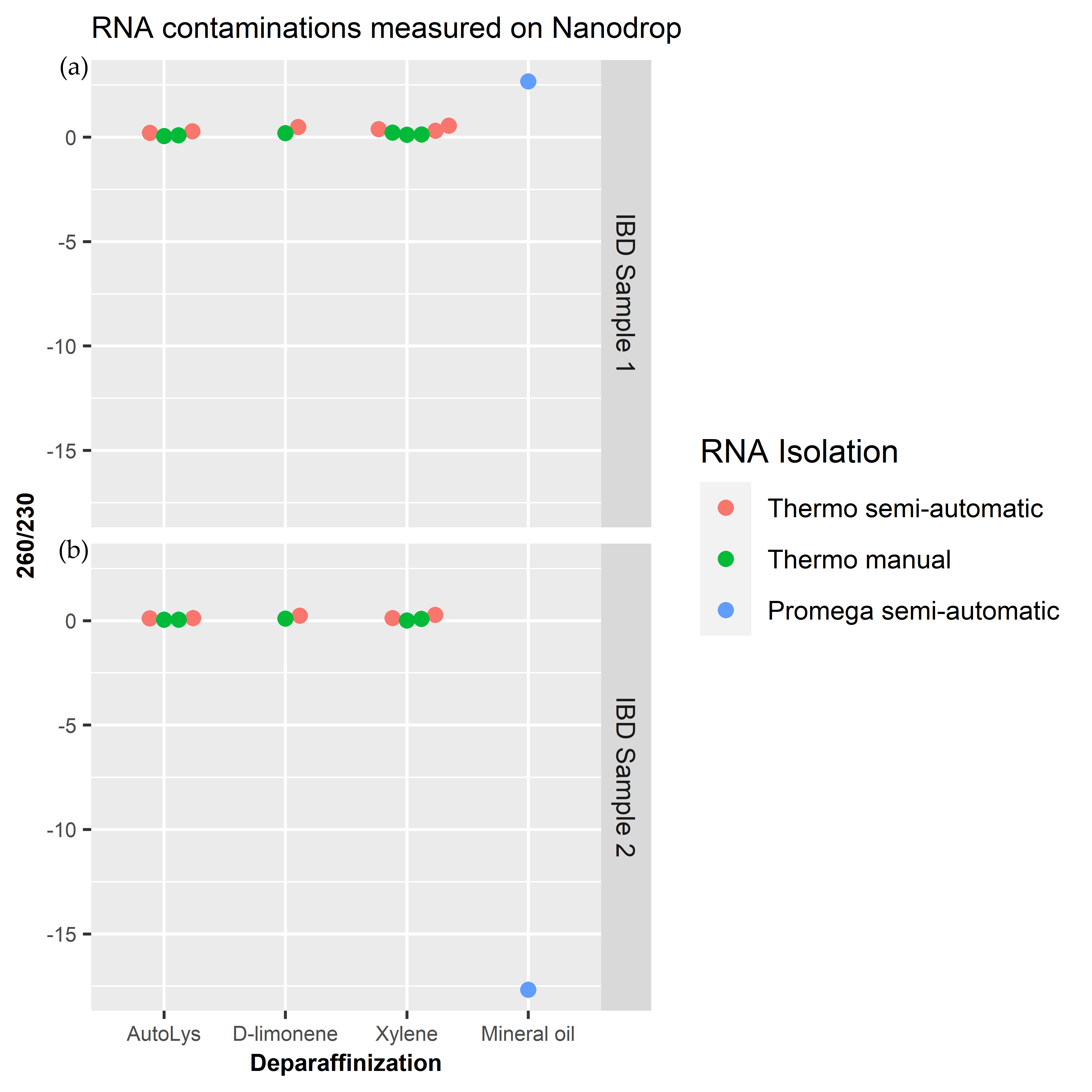

Supplement: Supplementary file 1 [file mps-07-00101-s001.zip › FigureS1_edit.tif]
